# Secreted herpes simplex virus-2 glycoprotein G alters thermal pain sensitivity by modifying NGF effects on TRPV1

**DOI:** 10.1186/s12974-016-0677-5

**Published:** 2016-08-30

**Authors:** Jorge Rubén Cabrera, Abel Viejo-Borbolla, Antonio Alcamí, Francisco Wandosell

**Affiliations:** 1 Centro de Biología Molecular Severo Ochoa, Consejo Superior de Investigaciones Científicas—Universidad Autónoma de Madrid, Nicolás Cabrera 1, Campus de Cantoblanco, E-28049 Madrid, Spain; 2 Centro de Investigaciones Biológicas en Red de Enfermedades Neurodegenerativas (CIBERNED), Madrid, Spain; 3 Present address: Department of Microbiology and Immunology, Geisel School of Medicine, Dartmouth College, Hanover, New Hampshire 03766 USA; 4 Present address: Institute of Virology, Hannover Medical School, Carl-Neuberg Strasse 1, 30625 Hannover, Germany

**Keywords:** Herpes infection, Pain sensitivity, Inflammation, Signaling

## Abstract

Genital herpes is a painful disease frequently caused by the neurotropic pathogen herpes simplex virus type 2 (HSV-2). We have recently shown that HSV-2-secreted glycoprotein G (SgG2) interacts with and modulates the activity of the neurotrophin nerve growth factor (NGF). This interaction modifies the response of the NGF receptor TrkA, increasing NGF-dependent axonal growth. NGF is not only an axonal growth modulator but also an important mediator of pain and inflammation regulating the amount, localization, and activation of the thermal pain receptor transient receptor potential vanilloid 1 (TRPV1). In this work, we addressed whether SgG2 could contribute to HSV-2-induced pain. Injection of SgG2 in the mouse hindpaw produced a rapid and transient increase in thermal pain sensitivity. At the molecular level, this acute increase in thermal pain induced by SgG2 injection was dependent on differential NGF-induced phosphorylation and in changes in the amount of TrkA and TRPV1 in the dermis. These results suggest that SgG2 alters thermal pain sensitivity by modulating TRPV1 receptor.

## Introduction

Genital herpes is a common sexually transmitted disease (STD) caused mainly by herpes simplex virus type 2 (HSV-2) and, with lower incidence, by herpes simplex virus type 1 (HSV-1) [1]. Both viruses initially infect epithelial cells within the skin and the mucosa during primary infection. Following replication in epithelial cells, HSV reaches and infects free nerve endings (FNE) of sensory neurons, establishing latency in ganglia of the peripheral nervous system (PNS). Reactivation of HSV leads to production of infectious viral particles, which are anterogradely transported along the axons to the skin and mucosa, starting a new cycle of infection [2].

Primary HSV infection, reactivation, and shedding can be asymptomatic or proceed with clinically evident disruption of the skin and mucosa, causing papules and ulcers. HSV infection can damage or kill epithelial and neuronal cells [3, 4]. The degree of cell damage, together with the associated inflammatory response, will determine the severity of the pathology [1]. Nearly all patients suffering from genital herpes present with itching, burning, and pain, caused probably by an extensive inflammatory response [5, 6]. Pain is an unpleasant sensory experience associated with a noxious stimulus that serves as a defense mechanism [7]. It is conveyed to the spinal cord and the brain by specialized sensory neurons known as nociceptors. Each type of nociceptor expresses a subset of receptors that responds to tissue damage caused by chemical, mechanical, or thermal stimulation. These receptors are activated once the stimulus reaches a certain threshold that is considered harmful. However, “pain thresholds” can vary in physiological or pathological conditions. Inflammation is a well-studied scenario in which pain thresholds are reduced in such a manner that non-harmful stimuli can be interpreted as painful [8]. During inflammation, several factors are secreted by damaged tissue and/or by immune cells that regulate nociceptors, decreasing the threshold of pain receptors.

Nerve growth factor (NGF) is a neurotrophic factor that belongs to the family of the neurotrophins [9]. NGF binds to and activates tyrosine kinase receptor TrkA to promote neuronal survival, axonal growth, and guidance in the PNS. NGF is also crucial for the development and maintenance of nociceptors [10]. At birth, the majority of nociceptors express TrkA. Afterwards, half of the nociceptive neurons downregulate the expression of TrkA reaching complete extinction during the first 3 weeks of life [11, 12]. In mature nociceptors, expression of TrkA is associated with peptidergic neurons expressing inflammatory neuropeptides like calcitonin gene-related peptide (CGRP) or substance P [13, 14]. This, together with the increased secretion of NGF during inflammation and its role in activating mast cells and neutrophils, underlines NGF’s role in inflammatory pain. Therefore, NGF coordinates pain and inflammation through the regulation of immune and neuronal cells [15–17].

The relationship between NGF and inflammatory pain has been well characterized at the molecular level. The thermal pain receptor transient receptor potential vanilloid 1 (TRPV1) is a non-specific cation channel activated by physical stimuli such as high temperatures and chemical stimuli like low pH or capsaicin. TRPV1 activation in nociceptive neurons leads to a painful and burning sensation [18]. TRPV1 is extremely regulated, and its threshold for activation is high (i.e., temperatures higher than 42 °C). However, under physiological or pathological conditions, activation thresholds can vary [18]. TRPV1 levels in peripheral nerves in the skin are low while levels in the cell bodies within the dorsal root ganglia (DRG) are high [13]. The NGF-TrkA axis is one of the most important regulators of TRPV1 amount, spatial distribution, and activation threshold [19, 20]. Inflammation of peripheral tissues promotes a local upregulation of NGF [21]. As a consequence, phosphorylation levels of TrkA are increased, affecting TRPV1 in two different ways. First, in the short term (from minutes to a few hours), TRPV1 is rapidly and locally phosphorylated in serine/threonine and tyrosine residues. Phosphorylation in serine/threonine residues decreases TRPV1 activation threshold [22–24], while phosphorylation in tyrosines alters TRPV1 subcellular localization from vesicles to the plasma membrane [20]. As a result of both increased phosphorylations in TRPV1, sensory neurons show a higher heat pain sensitivity in the short term. Second, in the long term (from hours to days), once NGF-TrkA complex has been retrogradely transported to the cell bodies, nociceptive neurons mobilize TRPV1 anterogradely, increasing its amount in nerve endings [19]. Furthermore, there is an increase in TRPV1 translation, but not expression, in nociceptive neurons [19]. Both mechanisms result in an increased heat pain sensitivity and hyperalgesia in the long term. Then, NGF secretion from damaged tissue or immune cells contributes to the burning and painful sensation at the site of inflammation through these mechanisms (for review, see [25, 26]).

We have recently shown that secreted glycoprotein G from HSV-2 (SgG2) binds NGF and alters NGF-dependent TrkA activation. SgG2 increases NGF-mediated axonal growth, blocking retrograde transport of TrkA, resulting in an accumulation of high levels of phosphorylated TrkA at the nerve endings. This could attract TrkA+ nerve endings to the site of infection [27]. However, since NGF is not only a neurotrophic factor but also an inflammatory mediator, we hypothesized that SgG2 could play a role in pain and burning sensation produced by HSV-2. Our present results show that injection of SgG2 in the mouse hindpaw increased thermal pain sensitivity at 3-h postinjection (hpi) but not at 16 hpi. At the molecular level, the effect induced by SgG2 at 3 hpi could be explained by an increased NGF-dependent TRPV1 phosphorylation in serine residues. We also found reduced amounts of TRPV1 at 16 hpi that may explain the lack of SgG2-increased thermal sensitivity at this time point. These results suggest that SgG2-NGF interaction alters thermal pain sensitivity, affecting the phosphorylation and spatio-temporal levels of TrkA and TRPV1 in a complex scenario.

## Results

### Injection of SgG2 results in transient enhancement of thermal pain sensitivity

**Fig. 1:**
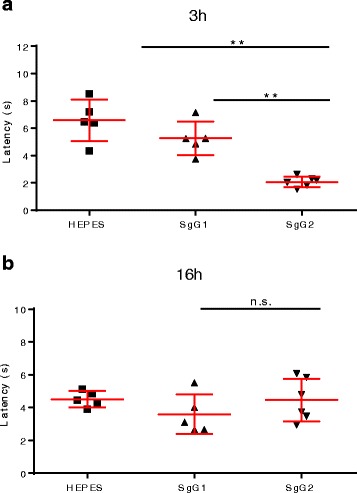
Injection of SgG2 induces a rapid and transient increase in thermal pain sensitivity. Graphs showing the latency time to withdraw the foot from an infrared lamp for non- or intradermally injected mice subjected to Hargreaves test at **a** 3 hpi or **b** 16 hpi. Viral proteins were injected in HEPES buffer which was also used as injection control. *Error bars* represent the mean plus standard deviation. ***p < 0.001; *n.s.* non-significant, *s* seconds

To test whether SgG2 could be responsible, at least partially, for the painful and burning sensation produced during clinical shedding of genital herpes, we injected SgG2 into the mouse hindpaw and performed a Hargreaves test (also known as plantar test) (Fig. 1). Injection of HEPES or a secreted version of glycoprotein G (SgG1) from HSV-1 did not result in any differential thermal sensitivity at 3 hpi (Fig. 1a). However, injection of SgG2 induced a statistically significant reduction in the latency time to withdraw the irradiated hindpaw at this time point compared to injection of HEPES (Mann Whitney test, *p* = 0.0043; unpaired *t* test with Welch’s correction, *p* = 0029), indicating that SgG2 increases thermal pain sensitivity (Fig. 1a). We repeated the test at 16 hpi in the same animals. At this time postinjection, all injected animals showed shorter latency responses compared probably due to a mild inflammatory process. Surprisingly, there were no differences between the injection of HEPES, SgG1, or SgG2 at this time point (Fig. 1b). More surprisingly, SgG2-injected mice at 16 hpi showed a higher latency period than SgG2-injected mice at 3 hpi (Mann Whitney test, *p* = 0.0022; unpaired *t* test with Welch’s correction, *p* = 0074). These results suggest that injected SgG2 increases heat sensitivity in mice only shortly after injection.

### SgG2 increases NGF-mediated TRPV1 phosphorylation on serine residues

**Fig. 2:**
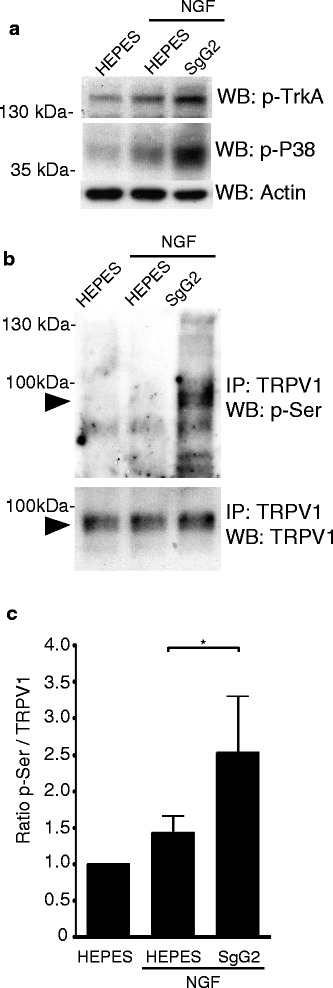
SgG2 increases NGF-dependent TRPV1 serine phosphorylation. DRG neurons were grown for 3 days in NGF medium, NGF starved for 16 h, and stimulated with HEPES, NGF in HEPES, or NGF plus SgG2 for 30 min. Western blots showing phosphorylation of TrkA and p38 (**a**) and TRPV1 phosphorylation in serine residues (**b**), which was detected following TRPV1 immunoprecipitation. **c** Graph showing the quantified serine phosphorylation in TRPV1. The data corresponds to the average of three independent experiments for TRPV1 serine phosphorylation. *Error bars* represent the mean plus standard deviation *p < 0.05

We have previously shown that SgG2 interacts with NGF and alters membrane localization, internalization, retrograde transport, and downstream signaling of TrkA. SgG2 could have an impact on sensory neurons expressing TrkA, including the regulation of heat pain sensitivity threshold. The best characterized heat pain receptor downstream the NGF-TrkA axis is TRPV1 [19, 20]. Stimulation of sensory neurons with NGF induces TRPV1 phosphorylation. To test whether SgG2 affects NGF-dependent TRPV1 phosphorylation, we used postnatal sensory neurons, which express TrkA and TRPV1 in higher percentage than adult sensory neurons. We starved dissociated mouse DRG neurons of NGF and exposed them to HEPES, NGF plus HEPES, or NGF plus SgG2. As a control, we analyzed the phosphorylation of TrkA and a downstream protein, P38. As previously described, NGF induced an increase in TrkA and P38 phosphorylation [19] (Fig. 2a). In agreement with our previous results [27], addition of NGF plus SgG2 resulted in higher phosphorylation of TrkA and P38 (Fig. 2a). To analyze TRPV1 phosphorylation status in this setting, we immunoprecipitated TRPV1 and detected phosphorylated serine and tyrosine residues by western blotting. SgG2 did not modify tyrosine phosphorylation of TRPV1 (not shown). However, the addition of SgG2 induced a statistically significant increase in serine phosphorylation of TRPV1 (Mann Whitney test, *p* = 0.0286; unpaired *t* test with Welch’s correction, *p* = ns, Fig. 2b, c). These results could explain the increased heat sensitivity promoted by SgG2 at 3 hpi, as increased serine phosphorylation of TRPV1 has been associated with reduced threshold to heat-related pain [22–24].

### Mobilization of TRPV1 to the dermis is reduced at 16 hpi of SgG2

**Fig. 3:**
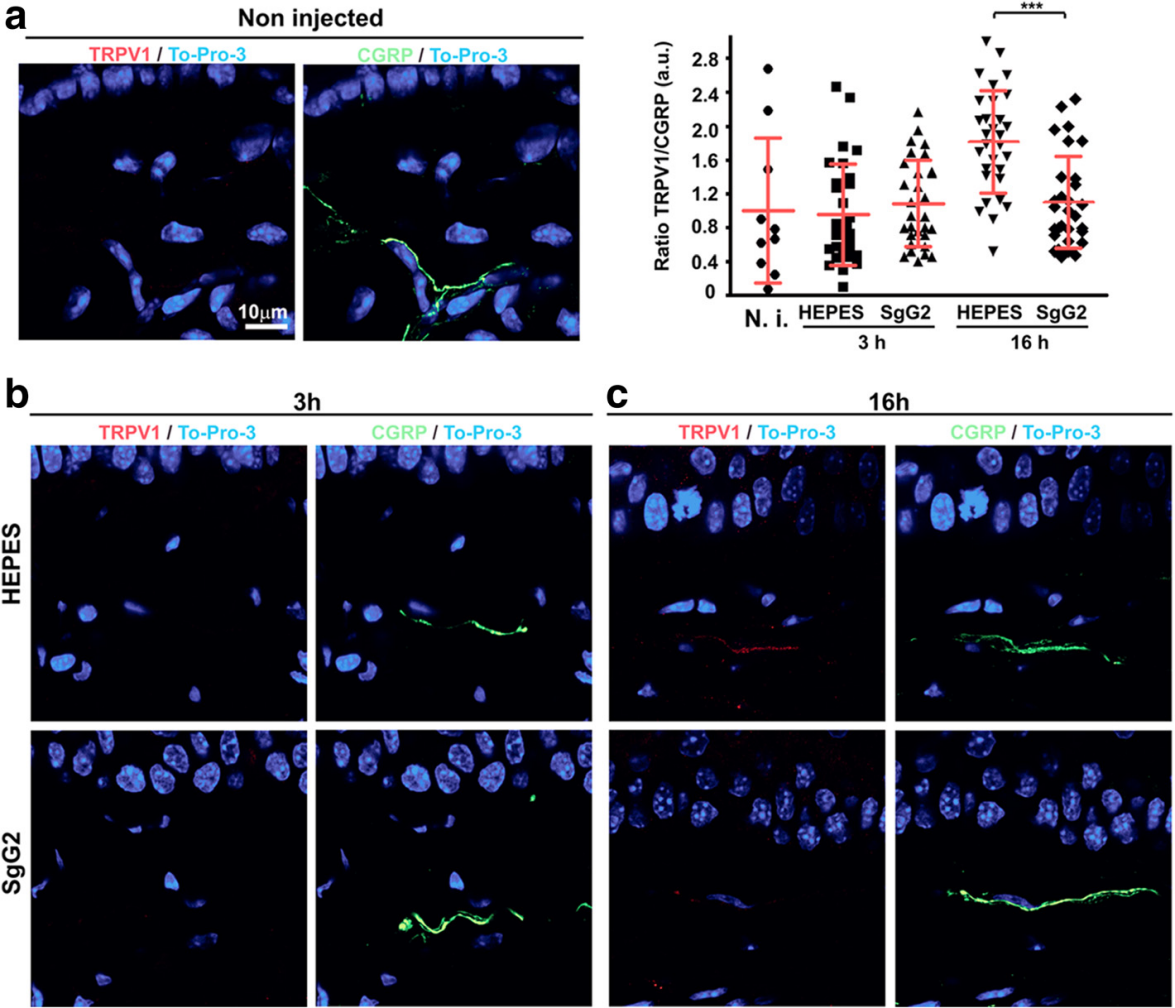
Injection of SgG2 blocks long-term transport of TRPV1 to the site of injection. Immunofluorescences showing the presence of TRPV1 and CGRP-positive nerves in the dermis of non-injected (**a**) or intradermally injected mice at 3 hpi (**b**) and 16 hpi (**c**). TRPV1 is shown in *red*, CGRP in *green*, and the cell nuclei are stained with To-Pro-3 and shown in *blue*. The graph represents TRPV1 quantification relative to CGRP in 10 random fields of each analyzed mouse (three mice injected). *Error bars* show the mean plus standard deviation. *a.u.* arbitrary units. ***p < 0.001

NGF plays a relevant role on TRPV1 phosphorylation and mobilization from DRG soma to nerve endings [13, 19]. Moreover, NGF increases the total amount of TRPV1 [19]. We observed an increase in heat pain sensitivity only at 3-h but not at 16-h post-SgG2 injection when NGF-dependent TRPV1 mobilization is predicted to start being significant, contributing to inflammatory heat increased sensitivity. The effect observed at 3-h post-SgG2 injection correlates with higher serine phosphorylation levels of TRPV1. The lack of effect on heat sensitivity at 16-h post-SgG2 injection prompted us to investigate the localization of TRPV1 in the injected tissue. As we performed intradermal injections, we focused our attention in the dermis, where the injected proteins should be present. It is described that the presence of TRPV1 in the dermis and epidermis is low in non-inflammatory conditions [19]. We also found that non-injected animals had very low levels of TRPV1 in the dermis (Fig. 3a). To detect nerves in the dermis, we used CGRP, a neuronal marker that has been associated with the expression of TRPV1 [13, 14]. We did not observe changes in the amount of TRPV1 in the dermis analyzing the injected area at 3 hpi in any of the experimental conditions (Fig. 3b). However, we observed a statistically significant increase in the presence of TRPV1 in the dermis of HEPES-injected mice at 16 hpi (Fig. 3c). This correlates with a tendency to reduce the latency time to withdraw the irradiated hindpaw between HEPES-injected mice at 3 and 16 hpi (Fig. 1). This could be due to a mild inflammatory response following injection of fluid in the hindpaw. Surprisingly, the amount of TRPV1 in the dermis of mice injected with SgG2 at 3 or 16 hpi was similar (Fig. 3c). The reduced amounts of TRPV1 in the dermis of SgG2-injected mice at 16 hpi compared to the HEPES control (Mann Whitney test, *p* < 0.0001; unpaired *t* test with Welch’s correction, *p* < 0.0001) could explain the absence of heat pain sensitivity despite increased levels of NGF-dependent TRPV1 serine phosphorylation. However, this result does not explain the lower mobilization of TRPV1 from cell bodies to nerve endings in SgG2 injected mice.

### SgG2 alters TrkA spatial distribution after injection

**Fig. 4:**
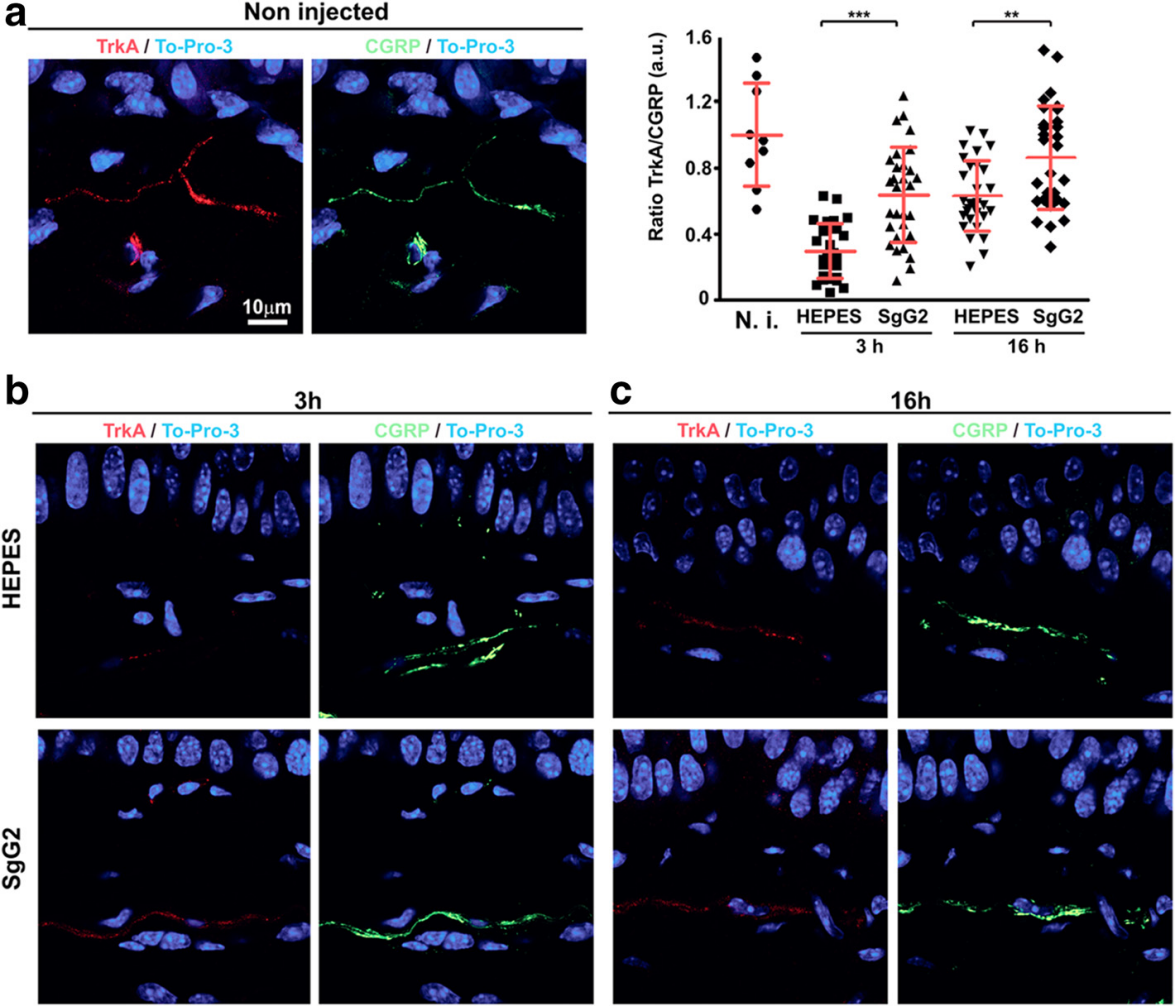
Injection of SgG2 blocks retrograde transport of TrkA. Immunofluorescences showing the presence of TrkA- and CGRP-positive nerves in the dermis of non-injected (**a**) or intradermally injected mice at 3 hpi (**b**) and 16 hpi (**c**). TrkA is shown in *red*, CGRP is shown in *green*, and the cell nuclei are stained with To-Pro-3 and shown in *blue*. The graph shows TrkA quantification relative to CGRP in 10 random fields of each analyzed mouse (three mice injected). *Error bars* represent the mean plus standard deviation. *a.u.* arbitrary units. **p < 0.01; ***p < 0.001

Sensory neurons have very long projections. Signals activated in a distal organ, like the skin, must reach the neuronal cell body for their processing. When NGF activates TrkA in distal tissues, TrkA must be endocytosed and retrogradely transported to the neuronal cell body for a complete response to NGF to occur [28]. Our previous results show that SgG2 impairs internalization and retrograde transport of TrkA in response to NGF [27]. Impairment of TrkA retrograde transport by SgG2 could explain the reduced mobilization of TRPV1 from cell bodies of DRG neurons to the nerve endings at 16-h post-SgG2 injection. To test if TrkA spatial distribution was altered, we analyzed the levels of TrkA in the dermis after injection (Fig. 4). As a control for nerves in the dermis, we used CGRP, a neuronal marker that has been associated with the expression of TrkA [11]. The levels of TrkA in the hindpaw dermis of non-injected animals were high (Fig. 4a). The levels of TrkA in the dermis of HEPES-injected mice were highly reduced at 3 hpi, probably due to NGF secretion by epidermal and immune cells following injection (Fig. 4b). However, injection of SgG2 resulted in lower reduction in the amount of TrkA in the dermis when compared to HEPES control at 3 hpi (Fig. 4b). The difference in TrkA levels was statistically significant between HEPES and SgG2 at this time point (Mann Whitney test, *p* < 0.0001; unpaired *t* test with Welch’s correction, *p* < 0.0001). We also measured the amount of TrkA at 16 hpi. At this time point, we observed that the level of TrkA started to be restored in the HEPES-injected dermis (Fig. 4c) but was still significantly lower than that in the dermis of animals injected with SgG2 (Mann Whitney test, *p* = 0.0022; unpaired *t* test with Welch’s correction, *p* = 0.0015) (Fig. 4c). These results suggest that TrkA spatial distribution is altered by SgG2 in vivo, remaining in the site of the injection which could explain why sensory neurons did not mobilize TRPV1 to the site of the SgG2 injection 16 h later.

## Discussion

HSV-1 and HSV-2 are two human pathogens with prevalence values around 65 % for HSV-1 [29] and 11.3 % for HSV-2 [30]. Following lytic infection of epithelial cells in the skin or the mucosa, they establish latency in peripheral ganglia. HSV-1 is more commonly acquired during childhood and is associated with establishment of latency in the trigeminal ganglia and oro-labial disease. HSV-2 is acquired later in life, normally through sexual contact, and is linked to establishment of latency in sacral ganglia and genital herpes. Genital herpes is a painful disease that can be caused by both HSV-1 and HSV-2. The symptoms (pain, itch, burning sensation) reported by HSV-1- and HSV-2-infected patients during the first episode of genital herpes are similar [5, 6]. However, periodicity and severity of genital herpes episodes increase when HSV-2 is the causative agent [1, 5].

The viral and cellular elements and the molecular mechanisms leading to burning sensation in HSV-2-induced genital herpes are not known. We show here that HSV-2 SgG induces heat-related pain, an effect that may contribute to HSV-2 pathogenicity. NGF is a neurotrophic factor involved in the development and maintenance of nociceptors [10] and an important mediator of inflammatory pain [17]. NGF is expressed in the mucosa and the skin, common sites of HSV replication during primary and recurrent infection [31]. We have recently described that SgG2 specifically binds NGF altering its receptor and downstream signaling pathways [27]. This results in increased neurite outgrowth and impairment of TrkA retrograde transport. On the contrary, SgG1 binds NGF but does not alter NGF activity [27]. TrkA, together with CGRP, is a common marker of peptidergic neurons present in the DRG. Since TrkA peptidergic neurons are enriched in the genitalia [32–34], we hypothesized that the modification of NGF/TrkA axis could have implications in the physiological properties of these nociceptors following HSV-2 infection. In particular, we hypothesized that SgG2 may be involved in HSV-2-induced pain during episodes of genital herpes.

HSV-2 infection, or transfection of SgG2, in the mouse footpad, results in a higher percentage of peptidergic FNE entering the stratum granulosum [27]. On the contrary, infection with HSV-1 or transfection of SgG1 does not affect peptidergic FNE growth [27]. In this report, we show that footpad injection of recombinant SgG2, but not SgG1, caused an increase in heat pain sensitivity at 3 hpi. This result correlates with increased phosphorylation of TRPV1 in serine residues after stimulation with recombinant SgG2 plus NGF. It also fits with previous data showing that TRPV1 serine phosphorylation is associated with reduced threshold activation and that some serine/threonine residues within the N and C termini of TRPV1 are implicated in receptor sensitization and activation [22–24, 35, 36].

Due to the long-term involvement of NGF in inflammation [17, 19] and the reports of chronic neuralgias induced by HSV-2 infection [37], we expected a prolonged effect of SgG2 inducing heat-related pain. However, SgG2 did not increase heat sensitivity compared to HEPES or other viral proteins at 16 hpi. At this time point, SgG2 injection induced less mobilization of TRPV1 to the site of injection than HEPES. This may explain the absence of differences in heat-induced pain at 16 hpi even with increased levels of TRPV1 serine phosphorylation. Reduced long-term mobilization of TRPV1 after SgG2 injection may appear contradictory. However, this result fits with our previous described data [27]. In order to accomplish all its biological functions during inflammation, NGF must be retrogradely transported from the inflamed distal tissue to the cell bodies of nociceptors [38]. Our previous results showed that SgG2 impairs NGF-induced TrkA retrograde transport in primary culture of neurons grown in microfluidic devices [27]. Similarly, we report here that injection of recombinant SgG2 alters TrkA spatial distribution of the CGRP^+^ neurons, maintaining high levels of TrkA in axons crossing the dermis, which would fit with a reduced TrkA retrograde transport. We hypothesize that this differential TrkA spatial distribution, with TrkA retained in the distal axons upon SgG2 intradermal injection, may explain our observations: in the short term, it may contribute to enhanced local TRPV1 phosphorylation, favoring an increase in heat pain sensitivity and, in the long term, it may explain the reduced mobilization of TRPV1 to the SgG2 injection site, diluting the short-term effect.

HSV-2 infection of genitalia can course from asymptomatic to extremely painful [1]. This suggests that HSV-2 interaction with the host is complex, and many different variables contribute to the final outcome. Then, understanding of SgG2 involvement in HSV-2-induced pain will require further studies in a more complete framework. Also, SgG2 interacts with chemokines and modulates chemokine receptor activity [39, 40]. Since chemokines also participate in nociceptive processes and inflammation [41], SgG2 could transiently contribute to pain induction by modifying chemokine activity. In conclusion, our results suggest that SgG2 alters thermal nociception by altering TrkA and TRPV1, and may contribute, at least partially, to HSV-2 induced pain.

## Materials and methods

### Ethics statement

All animal experiments were performed in compliance with national and international regulations and were approved by the Ethical Review Board of the Centro de Biología Molecular Severo Ochoa under the project number SAF2009-07857 and SAF2012-38957.

### Expression and purification of viral proteins

Viral proteins were expressed and purified by affinity chromatography from the supernatant of Hi-5 insect cells as previously described [40].

### In vivo injection of viral proteins in mouse hindpaw

All mice used were CD-1 males with 5 to 8 weeks of age from Charles Rivers (Wilmington, MA). Mice were anesthetized with a mixture of ketamine/xylazine (100 and 10 mg/kg body weight, respectively) prior to injection. We injected the viral proteins intradermally, in a region located between the proximal pads and heel of the ventral hindpaw. Always, the left hindpaw was injected; 5 μL of HEPES or indicated viral proteins at 6.8 μM in HEPES buffer were injected.

### Hargreaves plantar test

The Hargreaves test was performed using a standard apparatus from Ugo Basile (Monvalle, Italy). Mice were placed in a transparent acrylic box. A mobile infrared heat lamp was positioned to irradiate the left hindpaw. Intensity of the infrared heat lamp was set using non-injected mice. The latency time of the withdrawal response of each hindpaw was determined at 3- and 16-h postinjection. Measurements for each time point and mouse were taken several times and considered as technical replicates.

### Nerve staining and non-permeabilized inmunofluorescence

Mice were euthanatized and hindpaw skin was immediately removed by using a 3-mm biopsy punch and fixed in Zamboni’s fixative for 6 h. The biopsies were then washed, embedded in agarose sucrose, and sectioned using a vibratome; 50-μm, free-floating sections were washed in phosphate-buffered saline (PBS) with 0.5 % Triton X-100 (PBS + TX), blocked for 30 min in 10 % horse serum PBS + TX. Anti-CGRP (whole protein) antibody was from Sigma (St. Louis, MO), anti-extracellular TrkA AF1056 was purchased from R&D Systems (Minneapolis, MN), and anti-N-terminal TRPV1 (named as VR1, P-19) was from Santa Cruz (Santa Cruz, Ca). To-Pro-3 and secondary antibodies used were from Life Technologies (Life Technologies, Thermo Fisher Scientific, Carlsbad, CA). Confocal analysis was performed with a LSM 510 Confocal Laser Scanning Microscope from Carl Zeiss. Images for an experiment were taken with the same settings to allow proper comparison. Analysis and treatment of images was performed using LSM Image Browser, Fiji and Adobe Photoshop; firstly, a region of interest (ROI) in the CGRP image was defined. The area of the staining within this ROI was measured using Fiji after a threshold correction. The ROI was maintained for measurements in the other channels, and thresholds applied were the same for all the analyzed channels.

### Culture of dissociated DRG neurons

Ganglia were dissected from newborn mice (postnatal day 0–1), digested in collagenase and trypsin (Worthington, Lakewood, NJ), dissociated by trituration and plated on dishes previously coated with polylysine (250 μg/mL)-laminin (10 μg/mL) in DMEM-F12 (all three from Life Technologies, Thermo Fisher Scientific) containing 10 ng/mL NGF (Alomone labs, Jerusalem, Israel), 5 % horse serum, and 5 ng/mL of aphidicolin (A.G. Scientific, San Diego, CA) for 3 days.

### Treatment of DRG neurons

Dissociated neurons were grown during 3 days in vitro (DIV) and starved of NGF during 16 h when indicated. NGF and SgG2 were mixed in DMEM-F12 prior stimulation. To calculate NGF molarity, we considered NGF as a dimer (26 kDa). The concentrations used were 0.5 nM NGF with 100 nM SgG2 for signaling experiments, and the stimulation period was 30 min.

### Western blot and immunoprecipitation

Antibodies to detect p-TrkA Tyr490 (#9141) and p-p38 (Thr180, Tyr 182 #9211) were obtained from Cell Signaling (Danvers, MA). Anti-phospho-serine antibody (Ab 1603) was from Merck-Millipore (Darmstadt, Germany). To detect N-terminal actin, we used an antibody from Sigma (A-2228). For immunoprecipitation, 100 μg of DRG neuron extract was incubated overnight with anti-TRPV1 (termed VR1 P-19, Santa Cruz) antibody. Then, the mix was incubated with protein G-coupled agarose beads (GE Healthcare Waukesha, WI) and washed three times with lysis buffer (1 % NP40, 50 mM Tris pH 7.5, 150 mM NaCl and protease, and phosphatase inhibitors) prior to analysis by SDS-PAGE and western blotting.

### Statistical analysis

The significant value (*p* value) was calculated using GraphPad Prism. First, we calculated whether the data followed a Gaussian distribution using D’Agostino and Pearson omnibus normality test, Shapiro-Wilk normality test, and Kolmogorov-Smirnov normality test. Since the data did not follow a Gaussian distribution, we employed two different statistical analyses: Mann Whitney test and unpaired *t* test with Welch’s correction.

## Abbreviations

CGRP: Calcitonin gene-related peptide
DMEM-F12: Dulbecco’s modified Eagle medium with nutrient mixture F-12
FNE: Free nerve endings
HEPES: (4-(2-Hydroxyethyl)-1-piperazineethanesulfonic acid buffer
HSV-1: Herpes simplex virus type 1
HSV-2: Herpes simplex virus type 2
NGF: Nerve growth factor
PBS: Phosphate-buffered saline
PBS+TX: Phosphate-buffered saline containing Triton X100 detergent
PNS: Peripheral nervous system
SgG1: Glycoprotein G from HSV-1
SgG2: Secreted glycoprotein G from HSV-2
STD: Sexually transmitted disease
TrkA: Tyrosine kinase receptor for NFG
TRPV1: Thermal pain receptor transient receptor potential vanilloid 1
